# 

**Published:** 1904-07-09

**Authors:** 


### NOTICE TO CORRESPONDENTS.

All MSS., Letters, Books for Review, and other matters intended for the Editor should be addressed The Editor, "The Hospital," The Hospital Buildings, 28 & 29 Southampton Street, Strand, W.C.

The Editor cannot undertake to return rejected MS., even when accompanied by stamped directed envelope.

**Notice to Advertisers and Subscribers.**

All Advertisements, Orders for Copies of The Hospital, and Business Communications should be addressed to **THE MANAGER (Not to the Editor),** The Hospital, 28 & 29 Southampton Street, Strand, London, W.C.

The Cost of Subscription, post free, is as follows:—Per week, 3½d.; per quarter, 3s. 3d.; per half-year, 6s. 6d.; per annum, 13s. Foreign and Colonial:—Per annum, 19s. 6d., payable, in all cases, in advance.

**NOTICE.—Vol. XXXV. of "The Hospital" (October 1903 to March 1904), in handsome cloth binding, is now on sale at the office of this Journal, price 10s. 6d. Cases for binding the Numbers contained in this Volume 2s. Index to Vol. XXXV., 3½d., post free.**

**The Monthly part for July is now ready. Price 1s., by post, 1s. 3d.**

THE Metropolitan Provident Medical Association carries on its excellent work in so quiet a manner that comparatively few people know of its existence, and fewer still recognise in the principles it practises a very possible and reasonable solution of the "out-patient" difficulties which attend the present system of hospital relief. The association assists the establishment of provident dispensaries throughout the metropolis. The association has amongst its members 30,000 poor people who contribute towards their treatment and medicine supplied. Thus the system does not pauperise, and it is unattended by the inevitable waiting which must be experienced by patients attending the large out-patients' departments of the hospitals. The object is an excellent one for charity, as the money subscribed enables new dispensaries to be opened, which very soon become self-supporting.

### MEDICAL APPOINTMENTS.

**Ernest J. Beards**, M.R.C.S., L.R.C.P.Lond., Public Vaccinator, Parish of Balsall Heath, King's Norton Union. **A. Cruickshank**, M.B., C.M.Aberd., Certifying Factory Surgeon for the Stonehaven District, Kincardineshire. **C. V. Dingle**, M.D.Durh., B.Hy., D.P.H., Medical Officer of Health, River Tees Port Sanitary Authority. **D. A. R. Farquharson**, M.B., C.M.Aberd., Certifying Factory Surgeon for the Washington District, Durham. **R. Herd**, B.A., B.C.Camb., L.S.A., Medical Officer of Health, Heysham, Urban District. **F. W. Johnson**, M.B., Ch.B.Vict., Certifying Factory Surgeon for the Bawtry District of the Counties of York and Nottingham. **J. F. Keenan**, M.B., B.Ch.R.U.I., Certifying Factory Surgeon for the Balliualee District, Longford. **P. J. Kerwin**, L.R.C.P. & S., Surgeon to the Hospital and Government Medical Officer, Port Douglas, North Queensland. **John Lentaigne**, F.R.C.S.I., Medical Referee under the Workmen's Compensation Acts for the county and city of Dublin. **George A. Mavor**, M.A., M.B., Ch.B.Aberd., Resident Medical Officer to the Aberdeen City (Fever) Hospital, vice William E. Taylor, M.B., Ch.B., resigned. **G. Maw**, M.R.C.S., L.R.C.P., Certifying Factory Surgeon for the Ulceby District, Lincolnshire. **F. V. H. Mossman**, L.S.A., District Medical Officer of the Wortley Union. **R. G. Nesbitt**, L.R.C.P.&S.Irel., Medical Officer of Health, St. Just Urban District. **John Herbert Parsons**, B.S., D.Sc., F.R.C.S., Ophthalmic Surgeon to the Hospital for Sick Children, Great Ormond Street. **H. Vaughan Pryce**, F.R.C.S.Eng., M.A., M.B., B.C.Cantab., Honorary Medical Officer to the Brighton, Hove, and Preston Dispensary. **T. Sydney Short**, M.D.Lond., M.R.C.P., D.P.H.Cantab., Honorary Physician to the General Hospital, Birmingham. **Lilian G. Simpson**, M.D.Brux., L.R.C.P.&S.Edin., Senior Resident Medical Officer to the Canning Town Medical Mission Hospital and Dispensary. **R. J. Waugh**, M.R.C.S L.R.C.P., Third Resident Assistant Medical Officer, Bethnal Green Parish Infirmary. **W. Williams**, M.A., M.D., Examiner in Preventive Medicine in the University of Oxford. **J. S. Wilson**, M.B., C.M.Aberd., Medical Officer of Health, Brierfield Urban District.

**"The Hospital" Institutional Library.**

**"Hospitals and Asylums of the World." 4 Vols., with a Portfolio of Plans, complete £12 12s.**
**"Burdett's Hospitals and Charities, 1904." 5s. net.**
**"Burdett's Official Nursing Directory." 8s. net.**
**"Cottage Hospitals, General, Fever, and Convalescent." 10s. 6d.**
**"Uniform System of Accounts for Hospitals and Public Institutions." New Edition. 4s. net.**
**"Hospital Expenditure: The Commissariat." 2s. 6d.**
**"A Legal Handbook for the Use of Hospital Authorities." 2s. 6d.**
**"The Cottage Hospital Case Book or Register of Patients." 10s. and 12s. 6d.**
**All these are published by the SCIENTIFIC PRESS, Ltd., and may be obtained through any bookseller, or direct from the publishers, 28 and 29 Southampton Street, Strand, London, W.C.**

### A Dozen Books every Nurse should Possess.

| | | |
|---|---|---|
| I. | **A HANDBOOK FOR NURSES.** By J. K. WATSON, M.D. Profusely Illustrated. Cloth gilt. | 5/- net, 5/4 post free. |
| 2. | **A COMPLETE HANDBOOK OF MIDWIFERY.** By J. K. WATSON, M.D. Illustrated with 143 Diagrams. Cloth gilt. | 6/- net, 6/4 post free. |
| 3. | **PRACTICAL GUIDE TO SURGICAL BANDAGING AND DRESSINGS.** By WM. JOHNSON SMITH, F.R.C.S. Suitable size for the apron pocket. Profusely Illustrated. Cloth. | 2/- post free. |
| 4. | **SURGICAL WARD-WORK AND NURSING.** By ALEXANDER MILES, M.D.Edin. Second Edition. Illustrated. Cloth gilt. | 3/6 net, 3/10 post free. |
| 5. | **ART OF MASSAGE.** By MRS. CREIGHTON HALE. Second Edition Illustrated. | 6/- post free. |
| 6. | **ELEMENTARY ANATOMY AND SURGERY FOR NURSES.** By WM. McADAM ECCLES, M.S., M.B. Profusely Illustrated. Cloth. | 2/6 post free. |
| 7. | **ELEMENTARY PHYSIOLOGY FOR NURSES.** By C. F. MARSHALL, M.D, B.Sc., F.R.C.S. Profusely Illustrated. Cloth. | 2/- post free. |
| 8. | **NURSING IN DISEASES OF THE THROAT, NOSE, & EAR.** By P. MACLEOD YEARSLEY, F.R.C.S., M.R.C.S. Illustrated. Cloth. | 2/6 post free. |
| 9. | **OPHTHALMIC NURSING.** By SYDNEY STEPHENSON, M.B., F.R.C.S.E. New Revised Edition. Illustrated. | 3/6 net, 3/10 post free. |
| 10. | **THE EXAMINATION OF THE URINE.** By J. K. WATSON, M.D. Cloth boards. Suitable size for the apron pocket. | 1/- net, 1/1 post free. |
| 11. | **THE NURSES' DICTIONARY OF MEDICAL TERMS AND NURSING TREATMENT.** Compiled by HONNOR MORTEN. Fourth Edition. Suitable size for the apron pocket. Cloth. | 2/- post free. |
| 12. | **FEVERS AND INFECTIOUS DISEASES.** By WILLIAM HARDING, M.D.Ed. Bound in cloth. | 1/- post free. |

SEND FOR LATEST CATALOGUE OF NURSING MANUALS

*The above Books are obtainable of all Booksellers, or of*

**THE SCIENTIFIC PRESS, Ltd.,** 28 and 29 Southampton Street, Strand, London, W.C.

